# Antibody responses in Burkinabe children against *P. falciparum* proteins associated with reduced risk of clinical malaria

**DOI:** 10.3389/fimmu.2025.1521082

**Published:** 2025-02-26

**Authors:** Takaaki Yuguchi, Benedicta O. Dankyi, Rattanaporn Rojrung, Hikaru Nagaoka, Bernard N. Kanoi, Alfred B. Tiono, Issa Nebie, Alphonse Ouedraogo, Kazutoyo Miura, Jetsumon Sattabongkot, Sodiomon B. Sirima, Takafumi Tsuboi, Eizo Takashima

**Affiliations:** ^1^ Division of Malaria Research, Proteo-Science Center, Ehime University, Matsuyama, Japan; ^2^ Centre for Malaria Elimination, Institute of Tropical Medicine, Mount Kenya University, Thika, Kenya; ^3^ Groupe de Recherche Action en Santé (GRAS), Ouagadougou, Burkina Faso; ^4^ Laboratory of Malaria and Vector Research, National Institute of Allergy and Infectious Diseases, National Institutes of Health, Rockville, MD, United States; ^5^ Mahidol Vivax Research Unit, Faculty of Tropical Medicine, Mahidol University, Bangkok, Thailand; ^6^ Division of Cell-Free Sciences, Proteo-Science Center, Ehime University, Matsuyama, Japan

**Keywords:** *Plasmodium falciparum*, blood-stage, serology, wheat germ cell-free system, AlphaScreen, Burkinabe children, immune response, immunoglobulin G

## Abstract

Individuals residing in malaria-endemic regions with high disease transmission can develop semi-immunity within five years of age. Although understanding the target of the IgGs in this age group helps discover novel blood-stage vaccine candidates and serological markers, it has not been well elucidated due to limited accessibility to plasmodial antigens and samples. This study presents the first comprehensive analysis of antibody levels in plasma obtained from Burkinabe children (n=80, aged 0 to 5 years) to 1307 *Plasmodium falciparum* proteins expressed by the eukaryotic wheat germ cell-free system. Antibody levels were measured by AlphaScreen. We found that 98% of antigens were immunoreactive. The number of reactive antigens by the individual was correlated with increasing age. The most significant increases in seroprevalence occur during the first 2 years of life. By correlating antibody levels and the number of clinical malaria during a 1-year follow-up period, we identified 173 potential protein targets which might be associated with clinical immunity. These results provide valuable insights into how children acquired semi-immunity to malaria in their early lives.

## 1 Introduction

Malaria is a critical disease, especially for children under 5 years of age, as children in this age group accounted for approximately 76% of estimated malaria deaths worldwide in 2022 (1). Developing naturally acquired immunity to malaria mitigates severe symptoms and ultimately leads to the absence of any clinical manifestations. Young infants are protected from clinical malaria by maternal IgG; however, infants around 6-9 months of age are most vulnerable to malaria after maternal antibodies wane (2). When the children grow in areas of high malaria transmission, they gradually develop protective immunity to malaria, with increased antibody repertoires and levels through repeated exposure (3). The clinical symptoms shift from severe to mild, then asymptomatic as they grow into adulthood (4). Moreover, research has demonstrated that transfusion of immunoglobulin G (IgG) purified from malaria-immune adults could significantly reduce parasitemia and fever in young children (5), suggesting IgG is a critical component for malaria protective immunity. Therefore, understanding the target of the humoral immune response to malaria can lead to the development of vaccines and serological markers of exposure, which can help alleviate the global burden of the disease.

To advance the knowledge of which target antigens contribute to protective antibodies, several large-scale immuno-epidemiological studies have explored antibody profiles in malaria-exposed individuals to identify targets of protective immunity (6–8). In these studies, recombinant *Plasmodium falciparum* proteins expressed in an *Escherichia coli*-based system and printed on a microarray chip were probed with malaria-exposed human sera (6–8). Although the strategy proved valuable in leveraging a few antigens as candidate vaccines, the low discovery efficiency could be attributed to the poor quality of proteins expressed by the bacterial system (9–11).

To address this important gap, we established the wheat germ cell-free protein synthesis system (WGCFS) for expressing recombinant plasmodial proteins. The proteins have higher immunoreactivities compared with proteins produced by *E. coli* (9, 12, 13). Recently, over a hundred merozoite antigens expressed mainly by mammalian Expi293 cells for serological analysis (14). However, preparing over a thousand recombinant proteins with the system seems challenging. Moreover, because malaria parasites merely express glycosylated proteins, WGCFS, which lacks glycosylation machinery, has the advantage of expressing plasmodial proteins; thus, in some cases, potential N-glycosylation sites need to be mutated to prevent artificial glycosylation (14).

Moreover, we have optimized the AlphaScreen system for immuno-profiling with WGCFS-expressed malaria proteins (15). Previous studies using this approach have extensively screened blood-stage proteins with human serum samples from malaria-endemic regions, identifying several promising vaccine-candidate antigens (16, 17) in children and young adults aged 6-20 years in Uganda (16) and adults in Mali (17). In the Ugandan study (16), IgG analysis against 1827 WGCFS-expressed recombinant *P. falciparum* proteins revealed a broader protein immunoreactivity of 51% (938/1827), which was higher than similar studies using bacterially-expressed protein microarrays (7, 8). Finally, 128 proteins comprising previously characterized vaccine candidates with known parasite expression, localization, and function and novel uncharacterized proteins were selected for further evaluation (16). These initial data proved that baseline antibody responses targeting specific *P. falciparum* antigens could differentiate prospectively defined clinical malaria outcomes, forming a logical foundation for the present study. Previous studies were conducted with individuals over 5 years old. Therefore, in this study, we focus on immune responses in children under 5 years old, who are at a greater risk of clinical malaria (4, 18).

In a Burkina Faso study (19), demographic data analysis revealed that children’s age is associated with clinical malaria episodes. The risk was highest among ages 0-2 years, consistent with previously described vulnerability associated with the loss of protection by maternal immunoglobulin in the first six months of life. 3-5 year old children experience more malaria incidence than individuals over 5 years old during the high transmission season, suggesting an acquisition of protective immunity. Therefore, in this study, we sought to characterize the antibody responses in the plasma of children under 5 years old obtained from this Burkinabe cohort (19). This study spotlighted how people residing in malaria endemic areas acquired antibodies due to multiple *P. falciparum* infections in their early stages of life.

## 2 Materials and methods

### 2.1 Plasma samples and ethical statements

Burkinabe plasma samples from the reported study (19) were obtained from young children aged 0-5 years in the Saponé health district of Bazèga province, Burkina Faso. In 2007, both active and passive surveillance, along with cross-sectional surveys during low and high transmission seasons, were conducted throughout the year. Active surveillance was carried out by visiting the children’s homes twice a week. In the passive surveillance, parents in the cohort were encouraged to seek medical care whenever their child felt sick. Enrollment took place during the low transmission season, from January to February 2007, and the last observation was in February 2008 after the high transmission season, ranging from 355 - 401 days. This region was known as a high malaria transmission area, with an entomological inoculation rate (EIR) estimated at 44.4 per month during the rainy seasons from June to September. The majority of the population relies on subsistence agriculture, cultivating millet and raising domestic animals. Houses are generally constructed with mud walls and roofs made of either grass or corrugated iron. The population under demographic surveillance system had less than 5% bed net coverage in that year, which means that nighttime conditions favorable for *Anopheles* mosquitoes to feed were likely consistent. The cross-sectional surveys of the active surveillance cohort during the high transmission season reported that age did not show a consistent relationship with the prevalence of asymptomatic *P. falciparum* infection: 37.6% for the 0-1 year-age group, 53.8% for the >1-2 year-age group, 47.1% for the >2-3 year-age group, 54.5% for the >3-4 year-age group, 52.9% for the >4-5 year-age group. A clinical malaria episode is defined as a fever (>37.5°C) or a history of fever in the last 24 hours accompanied by a malaria-positive smear confirmed by microscopic examination. The confirmed uncomplicated malaria cases were treated with Coartem® or Artesunate Amodiaquine. After recording an episode, children underwent a 28-day censoring period to ensure the infection responsible for the episode was recorded only once. Written informed consent was obtained from each child’s parent or legal guardian before the study commenced. This study was conducted in compliance with the International Conference on Harmonization of Good Clinical Practices, the Declaration of Helsinki, and the regulatory requirements of Burkina Faso (19). All required ethical approvals were obtained from the Burkina Faso Ethics Committee for Health Research (ID: DMID No. 06-0020). Plasma samples from anonymized healthy donors from Bangkok, a malaria non-endemic area, were obtained from the Thai Red Cross (TRC) with broad consent and used as the negative control. The protocol for using the plasma samples in this study was approved by the Institutional Review Board of Ehime University Hospital, Japan (Aidaiibyourin 1507005).

### 2.2 Production of *P. falciparum* proteins and measurement of IgG levels with AlphaScreen system

This study used a genome-wide protein library consisting of 1307 recombinant proteins derived from 1207 genes of *P. falciparum* strain 3D7 (16) (**Supplementary Table S1**). Each protein was annotated by PlasmoDB (http://www.plasmodb.org). The recombinant proteins were expressed by the WGCFS and were mono-biotinylated by BirA, with the semi-automated GenDecoder 1000 robotic protein synthesizer (CellFree Sciences, Matsuyama, Japan) as previously described (16). The AlphaScreen was performed to profile the *P. falciparum* antigen-specific IgG antibodies (16, 20–22). All the liquid handling procedures were semi-automated by VIAFLO 384 and VIAFLO ASSIST (Integra Biosciences, Zizers, Switzerland). A mixture of 5 μL of 50-fold diluted non-purified biotinylated recombinant *P. falciparum* protein and 10 μL of 4000-fold diluted plasma in the reaction buffer (100 mM Tris-HCl pH 8.0, 0.01% [v/v] Tween-20, and 0.1% [w/v] bovine serum albumin) was incubated in 384-well OptiPlate microtiter plates (PerkinElmer, Waltham, MA, USA) for 30 minutes at 26°C. Subsequently, 10 μL of a mixture of streptavidin-conjugated donor beads and protein G (Thermo Scientific, Waltham, MA, USA) conjugated acceptor beads (PerkinElmer) was added, and then incubated for 60 minutes at 26°C in the dark. The luminescence signal was detected at 620 nm using the EnVision plate reader (PerkinElmer). The wells containing serially diluted biotin-SP-ChomPure human IgG (Jackson ImmunoResearch Laboratories, West Grove, PA, USA) served as a standard curve to ensure day-to-day and plate-to-plate normalization. This generated normalized AlphaScreen counts using a 5-parameter logistic standardization curve. Plasma samples were randomized to minimize experimental bias.

The Burkina Faso cohort samples were tested in singlicate well in one assay, whereas a pooled plasma sample from TRC (negative control plasma) were tested in singlicate well in three assays. The seropositivity cutoff values were defined at the negative control plasma’s mean plus two times the standard deviation (mean + 2SD) in log-transformed values. Proteins were regarded as immunoreactive if more than 10% of the volunteers had the log-transformed normalized AlphaScreen counts above this cutoff level and applied to subsequent statistical analysis.

### 2.3 Statistical analysis

Statistical analyses were performed using R software, version 4.3.2 (23). The values referred to as ASC throughout this paper were defined by subtracting the log-transformed average TRC antibody response for each antigen, considered as the background, from the log-transformed normalized AlphaScreen counts for each individual sample. Any values resulting from this subtraction that were below zero were treated as zero. The Wilcoxon rank sum test was utilized to assess the statistical differences in median seroprevalence among age groups. The p-values obtained from this analysis were adjusted with Bonferroni correction. The Spearman’s rank correlation test was used to evaluate the correlation between age and the number of episodes per period of malaria-risk (RpT), and between ASC and RpT for 79 individual plasma samples. The 95% confidence interval of the Spearman’s rank correlation was computed using 1,000 bootstrapping iterations, sampling 100% of the original dataset each time. The period of malaria risk was defined by subtracting the number of episodes times 28 days of the censoring period from the duration of the surveillance (ranging from 355 - 401 days), that is to say, an increasing number of episodes leads to a rising RpT. The Welch’s t-test evaluated the differences in average ASC for each antigen between the 0-0.5 and >0.5-1 age groups.

## 3 Results

### 3.1 Characteristics of the plasma samples

In 2007, both active and passive surveillance were conducted in Burkina Faso over approximately one year. This region had been harboring an underdeveloped healthcare system for malaria prophylaxis. Two cohorts of children, each aged 0-5 years, were evaluated for malaria incidence to understand the epidemiology of malaria for future vaccine and drug trials (19). The total of 311 plasma samples were collected at the beginning of the cohorts, during the middle of the low transmission season, and divided into six age groups: 0-0.5, >0.5-1, >1-2, >2-3, >3-4, and >4-5 years. In this study, we examined 80 out of 311 plasma to ensure a balanced number of samples and gender ratio in each age group (**Table 1**). Basically 14 samples were randomly selected from each age group (>0.5 year old groups), excluding those that had recorded no clinical malaria episodes (24), ensuring that the gender ratio was as equal as possible among the age groups. For the 0-0.5 age group, all plasma samples were included regardless of the number of clinical malaria episodes and gender. The median age of the used samples was 2.11 years, with a range from 0.23 to 4.88 years. The male participation rate was 56%. The number of malaria incidents had a median of 3, with a range from 0 to 6 (**Table 1**). The number of episodes per period of malaria risk (RpT) ranged from 0 – 0.029.

**Table 1:** Characteristics of plasma samples in this study.

Age group	0-0.5 (n = 10)	>0.5-1 (n = 14)	>1-2 (n = 14)	>2-3 (n = 14)	>3-4 (n = 14)	>4-5 (n = 14)	All (0-5) (n = 80)
Age at sampling, median (range)	0.40 (0.23 – 0.46)	0.81 (0.52 – 0.97)	1.50 (1.01 – 1.90)	2.36 (2.01 – 2.98)	3.42 (3.06 – 3.99)	4.69 (4.07 – 4.88)	2.11 (0.23 – 4.88)
Gender (Male %)	70	71	50	50	50	50	56
Number of episodes, median (range)	3 (0 - 6)	2.5 (1- 5)	4 (1- 6)	3 (1- 5)	1.5 (1- 6)	2 (1- 4)	3 (0 - 6)
Seroprevalence, median (IQR)	50 (30-60)	64 (43-79)	79 (57-93)	71 (57-86)	79 (50-93)	86 (71-93)	73 (54-85)

### 3.2 Seroprevalence of human IgG responses

**Figure 1:**
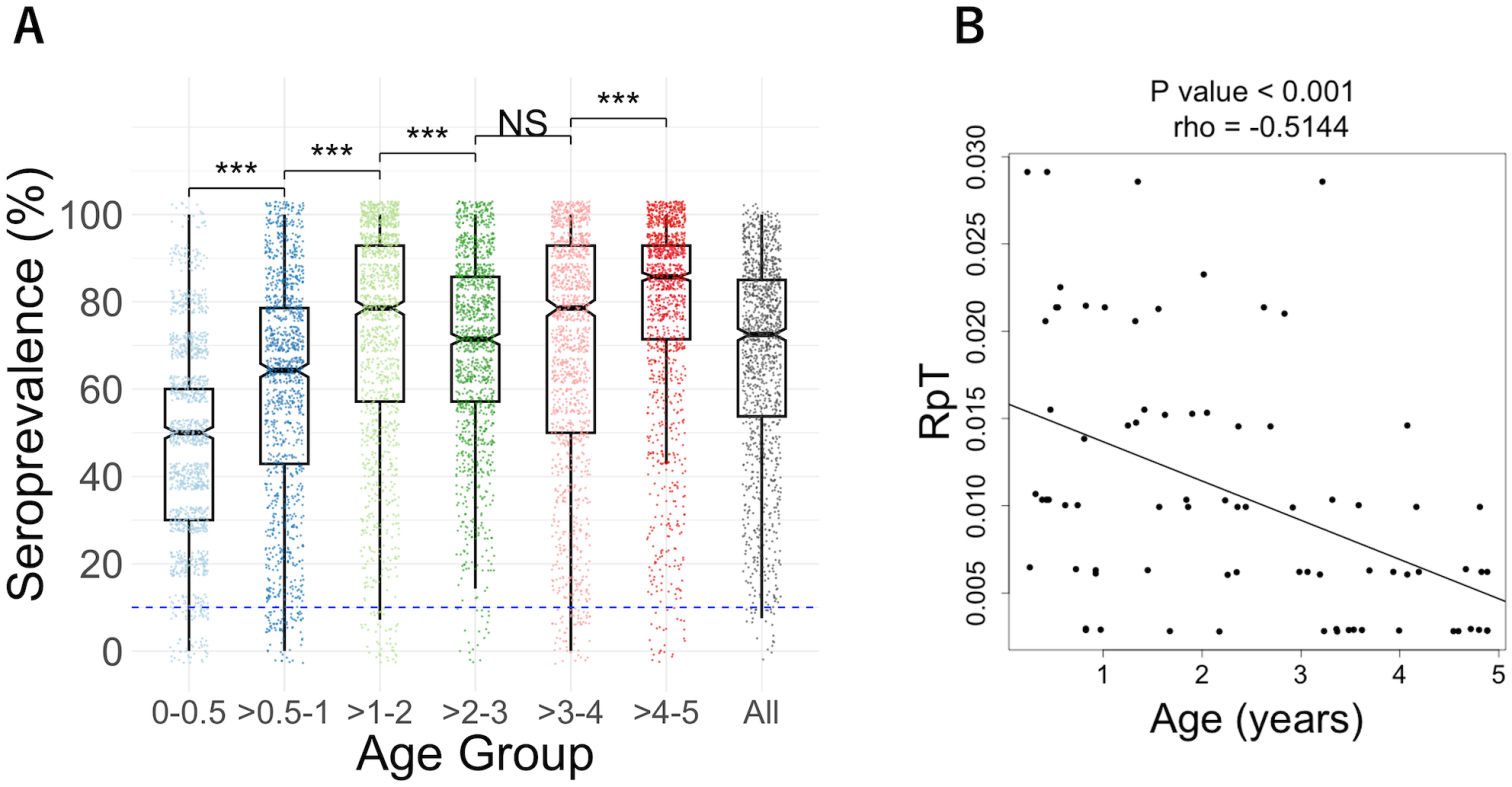
**(A)** Seroprevalence of each age group (0-0.5, >0.5-1, >1-2, >2-3, >3-4, >4-5, and all). Box plots illustrated the median and the 25/75 percentiles, with whiskers extending to 1.5 times the interquartile range. Dashed blue lines indicate the cut-off value of 10% seroprevalence. The differences among age groups were analyzed by the Wilcoxon rank sum test with Bonferroni correction: ***P<0.001, NS, not significant. **(B)** The relationship between age and the number of episodes per period of malaria risk (RpT). A significant negative correlation between age and RpT for 79 individual plasma samples (one plasma from a 0-0.5 group infant with RpT=0 was excluded) was observed using Spearman’s rank correlation, rho = - 0.514, P = 1.236×10^-6^. The lines were drawn by using linear regression.

To determine the immunoreactivity of parasite proteins, the antibody levels were measured using AlphaScreen (**Supplementary Table S2**), optimized for WGCFS-expressed proteins (16, 20–22). As a result, 98% of tested antigens were immunoreactive, i.e. antigens reacted with more than 10% of the plasma samples measured. The median seroprevalence was 73% (**Table 1**, **Figure 1A**). The number of reactive antigens by the individual was correlated with increasing age (Spearman’s rank correlation; rho = 0.299, p < 0.01), consistent with previous studies (25–27). The median of seroprevalence for each age group was as follows: 50% for the 0-0.5 year-age group, 64% for the >0.5-1 year-age group, 79% for the >1-2 year-age group, 71% for the >2-3 year-age group, 79% for the >3-4 year-age group, and 86% for the >4-5 year-age group. The largest increases were observed until 2 years old, i.e., the median of seroprevalence increased from 50% to 79% (**Table 1**, **Figure 1A**). The number of reactive antigens was not correlated with the subsequent number of clinical malaria episodes (rho = -0.074, p = 0.5166). Meanwhile, the relationship between age and the RpT among 79 sera, excluding plasma with recorded 0 episodes, showed a significant negative correlation (rho = -0.514, P = 1.236×10^-6^) (**Figure 1B**).

### 3.3 Potential IgG targets for clinical immunity

**Figure 2:**
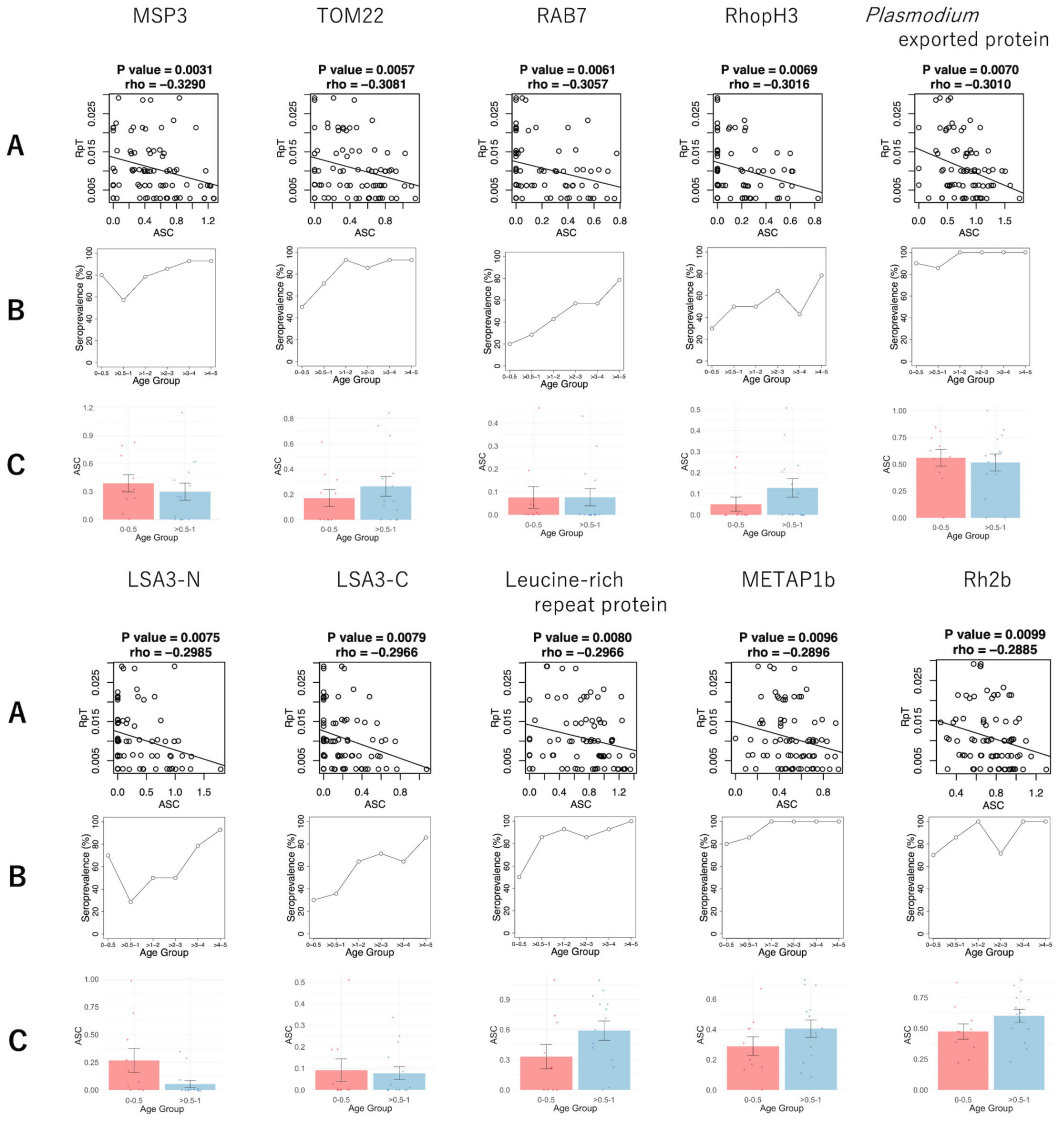
The antibody responses of the top 10 antigens associated with reduced risk of clinical malaria episodes. **(A)** The scatter plots of the ASC (normalized, log-transformed and backgound-subtracted AlphaScreen Count) and RpT (the number of episodes per period of malaria risk) with P-value and rho derived from Spearman’s rank correlation. The straight lines were drawn by linear regression. **(B)** The seroprevalence of different age groups. **(C)** The comparison of log-transformed ASC between the 0-0.5 and >0.5-1 age groups. The bar and error bar indicate the mean and the standard error, respectively. No significant differences were observed by Welch’s t-test.

To identify potential antibody targets that might be associated with the reduction of clinical malaria episodes, we conducted a correlation analysis between ASC and the RpT using 79 plasma samples. The analysis did not include a case under 0.5 years without malaria incidents during the follow-up period, which may have simply lacked exposure to malaria (24). Negative correlations between ASC and RpT were observed among the 173 antigens derived from 160 genes (unadjusted p < 0.05) before adjustment of the family-wise error rate (**Supplementary Table S3**). However, since a large number of antigens (1,307 proteins) were examined with relatively fewer samples (79 plasmas), the adjustment for multiple testing did not show significant antigens. The top 10 antigens (**Figure 2A**) ranked by the unadjusted p-values were MSP3, TOM22, RAB7, RhopH3, *Plasmodium* exported protein, LSA3-N (65-749 aa), LSA3-C (750-1433 aa), Leucine-rich repeat protein, METAP1b, and Rh2b (**Table 2**). Next, the immune responses to the top 10 antigens were examined individually. Seroprevalences of the antigens showed that antibody acquisition increased with age in general (**Figure 2B**). However, both *Plasmodium* exported protein and METAP1b were highly immunoreactive across all age groups.

**Table 2:** Top 10 antigens associated with reducing the risk of clinical malaria episodes.

Rank	Annotation	Gene ID	Rho	95% CI	P value
1	MSP3	PF3D7_1035400	-0.3290	(-0.52, -0.10)	0.0031
2	TOM22	PF3D7_0524700	-0.3081	(-0.49, -0.11)	0.0057
3	RAB7	PF3D7_0903200	-0.3057	(-0.51, -0.07)	0.0061
4	RhopH3	PF3D7_0905400	-0.3016	(-0.50, -0.09)	0.0069
5	*Plasmodium* exported protein	PF3D7_0702500	-0.3010	(-0.49, -0.09)	0.0070
6	LSA3-N (65 – 749 aa)	PF3D7_0220000	-0.2984	(-0.50, -0.07)	0.0075
7	LSA3-C (750 – 1433 aa)	PF3D7_0220000	-0.2966	(-0.50, -0.09)	0.0079
8	Leucine-rich repeat protein	PF3D7_1427900	-0.2965	(-0.49, -0.07)	0.0080
9	METAP1b	PF3D7_1015300	-0.2895	(-0.48, -0.08)	0.0096
10	Rh2b	PF3D7_1335300	-0.2884	(-0.47, -0.09)	0.0099

In order to evaluate the potential contribution of maternal antibodies to the top 10 antigens, we also compared the means of the ASC between the 0-0.5 and >0.5-1 age groups (**Figure 2C**). Antibodies against MSP3, *Plasmodium* exported protein, and LSA3-N showed higher mean ASC in the 0-0.5 years old compared to the >0.5-1 age group, consistent with seroprevalences (**Figure 2B**). However, no significant differences were found for any of these 10 antibodies.

## 4 Discussion

The goal of this study was to determine the antibody responses in children under the age of 5. We showed that the WGCFS/AlphaScreen is an advanced method for profiling antibodies against *P. falciparum* antigens (15, 16, 20–22). There is a lack of investigation into the antibodies present in children under 5 years old living in high malaria transmission areas, who acquired protective immunity against the parasite in their early life. Age is one of the major factors influencing the risk of clinical malaria in areas with high malaria transmission intensity (**Figure 1B**), as described in previous studies (28–30). The largest increases in seroprevalence were observed until 2 years of age. Specifically, the median seroprevalence increased from 50% in the >0-0.5 year age group to 79% in the >1-2 year age group (**Table 1**, **Figure 1A**). We also detected antibodies under 0.5 years old, presumably a combination of IgGs transferred from their mothers and acquired immune responses. While not statistically significant, some antibody levels appeared to be higher in 0-0.5 age group than those in >0.5-1 age group children. Further investigations, we intend to explore the function of the antibodies against the selected antigens to understand how people develop semi-immunity against the parasites. In addition, comparing antibody levels in children and their mothers would be valuable in understanding the role of maternally transferred IgG in malaria protection.

*P. falciparum* proteins are known to be difficult to express, which can be attributed to the characteristics of *P. falciparum* genes, including their remarkably high A/T content (~ 80%), codons that are infrequently used in heterologous expression systems, the enrichment of many proteins in repetitive sequences, most proteins being of high molecular weight (>60 kDa) with disordered regions and basic isoelectric points (>6.0), and multiple disulfide bonds in extracellular proteins (15). Proteins or peptides produced by *E. coli* cell-free systems are widely used in microarrays to detect immune responses to malaria. This technology can identify reactions to blood-stage proteins (8), proteome-wide proteins (all life stages) (31), and *P. falciparum* erythrocyte membrane protein 1 (PfEMP1) domains (32). However, a significant challenge with using proteins synthesized by the *E. coli* cell-free system is their low reactivity to sera from individuals infected with malaria, probably due to the poor quality of proteins (9–11). A recently developed protein microarray, KILchip v1.0, only contains a hundred merozoite antigens of *P. falciparum*, expressed mainly by mammalian Expi293 cells and has successfully identified potential markers of recent malaria exposure in areas with moderate transmission (14, 33). In both platforms, the adsorption process of proteins onto the microarray slide may influence protein structure and interactions, leading to the detection of lower than actual antibody responses (14, 34). Taken together, to compare antibody responses among different field settings head-to-head, it is essential to use identical technology platforms, i.e., protein expression and detection methodology.

In order to achieve this objective, we utilized WGCFS/AlphaScreen technology. Although we used non-purified proteins, previous studies have demonstrated that WGCFS/AlphaScreen technology is capable of measuring antibody levels with high specificity (15, 20). We compared the results with our previous studies using human samples obtained from various field settings. Our previous results with serum samples from children and young adults aged 6-20 years in Uganda have demonstrated 51% immunoreactivity against 1827 antigens (16). In general, children have less IgG reactivity against malaria antigens than adults in malaria-endemic areas (3). However, we revealed that 98% of antigens were immunoreactive against plasma samples from Burkinabe children from 0 to 5 years old. Thus, this study indicates that young children, even those under 5 years old, may show a greater number of IgG responses against malaria antigens than observed in the previous study (16). However, a direct comparison of the two studies is not easy. Earlier studies lacked a method for normalizing plate-to-plate variation with biotinylated IgG, resulting in high deviations in negative control values and an elevated cutoff value (16, 17). On the other hand, our recent studies utilizing the normalization method showed 97.5 to 100% immunoreactivity with blood-stage antigens, consistent with this study (22, 35). To clarify the relationship between age and immunoreactive antigens, it is necessary to evaluate antibody responses across a wide range of ages, from infants to adults, in the same exposure conditions using the same set of antigens in future studies.

This study indicates that antibody responses against 173 antigens might be associated with clinical protection using the Spearman rank sum correlation between ASC and RpT (**Supplementary Table S3**), while a future study with larger samples is required to confirm the results. The top 10 antigens (**Table 2**) included antigens such as MSP3, RhopH3, Rh2b, and LSA3, which have previously been shown that the antibodies had growth inhibitory assay (GIA) activities (17, 36–39). Among them, the IgG response against MSP3, expressed on the merozoite surface, showed the highest negative correlation with RpT. MSP3 is one of the most advanced asexual blood-stage vaccine candidates that has been tested in phase 2b trials as part of GMZ2, which is a combination of MSP3 and GLURP (Glutamate-rich protein) of *P. falciparum* (40). Our earlier studies, which utilized sera from different age groups and areas compared to those in this study, individuals aged 6 to 20 years from a high transmission region in Uganda (35) and adults from a low transmission region in Thailand (41), indicated that anti-MSP3 antibodies also play a role in protective immunity in both settings. Additionally, our recent study suggested that autoantibodies induced by the parasite infection also recognized MSP3 among the top-rank protective antigens (42). We observed the seroprevalence of at least 57% for MSP3 across all age groups (**Figure 2B**), suggesting anti-MSP3 IgG contributes to protection in children under five. The in-depth studies reported that IgG3 antibodies specific to MSP3 are associated with clinical malaria protection, using sera from people in hyperendemic areas, aged 0-15 or older in Myanmar (43), and aged 0-21 or older in Senegal (44). To investigate which IgG subclasses against MSP3 are responsible for our data needs to be investigated in the future.

The well-known merozoite rhoptry proteins, RhopH3, and Rh2b, are also identified among the top 10 antigens. Seroprevalence against RhopH3 starts at 30% in the youngest age group and increases with age to 78% in the oldest age group (**Figure 2B**). In contrast, seroprevalence against Rh2b reached 70% at the youngest 0-0.5 years (**Figure 2B**). These findings indicate that anti-Rh2b IgG is acquired much earlier in life compared to anti-RhopH3.

LSA3 is also identified among the top-ranked antigens. The LAS3 protein is expressed in both sporozoite surface and merozoite dense granules (17, 45). Our previous study using IgG samples from Malian adults indicated that anti-LSA3-C antibody levels were significantly correlated with the *in vitro* GIA activities (17). LSA3 antibodies have also been found in autoantibodies with GIA activities (42). Both the N- and C-terminal regions of LSA3 have been studied as vaccine candidates. Immunization of *Aotus* monkeys with a peptide from the 100-222 amino acid region in the N-terminal, which does not contain repeat elements, resulted in *in vivo* protection against sporozoite injection (46). Peptides designed to cover the entire LSA3 sequence have demonstrated a peptide, LSP10, located in a nonrepeat region of LSA3 (aa 893 - 999), associated with malaria protection in children aged 7-15 (47). LSA3 is frequently associated with malaria protection in serological approaches (17, 42, 47). Our results showed a progressively increasing seroprevalence with age for both N- and C-terminal regions (**Figure 2B**). Although LSA3 may have multiple protective regions, we should consider the possibility of cross-reactivity via the repeat regions of LSA3 itself or other antigens (48). Nonetheless, LSA3 remains a promising vaccine candidate.

It is possible that antibody responses, which show significant correlations with malaria risk, may not have a direct link with malaria protection (clinical immunity) in any epidemiology studies. However, these antibodies could be used as biomarkers to indicate the level of development of protection against malaria and/or the accumulation of malaria exposure, rather than being used as vaccines. Our results indicate that other top antigens could potentially be used as candidates for vaccines or as serological markers, but further studies are needed to understand their relationship to protection.

One limitation of our study is the small sample size (n=80), which may not fully represent general immune associations in the whole young children populations in the study area. In addition, the small sample size relative to the large number of antigens (n=1307) tested in this study did not allow for conducting a more appropriate analysis to reduce type I errors (e.g., adjustment with the False Discovery rate, conduct a linear regression analysis including an age factor). Furthermore, we only included one plasma sample from children under 3 months. As a result, comparing the mean ASC between the 0-0.5 and >0.5-1 age groups may be significantly impacted due to the wide range of IgG responses observed in children aged 3-23 months (49). To confirm our findings, further studies with larger sample sizes or research focusing on promising proteins are necessary. Also, the weaknesses of this study are the absence of data on antigenic polymorphisms in this region at the time of sample collection and the functional analysis of the child antibodies, both affecting the conclusion of this study. In future studies, we would like to explore the functional aspect of the antibodies and the influence of antigenic polymorphisms.

## 5 Conclusion

This study evaluated antibody responses against 1,307 *Plasmodium falciparum* proteins in Burkinabe children. The findings demonstrate the most significant increases in seroprevalence occur during the first 2 years of life. In addition, we identified potential IgG targets for clinical immunity. Once the target protein(s) are confirmed in a larger study, further research is warranted to elucidate the identified proteins, which could inform the development of novel malaria vaccines and serological markers.

## Data Availability

The original contributions presented in the study are included in the article/**Supplementary Material**. Further inquiries can be directed to the corresponding author.
